# Characterization of Digital Jaw Tracking Values in Prosthetic Rehabilitations: A Case Series

**DOI:** 10.3390/dj14070432

**Published:** 2026-07-13

**Authors:** Sofia Lobo, Vanessa Machado, Inês Argolinha, João Rua, José João Mendes, Junying Li, João Botelho

**Affiliations:** 1 Egas Moniz Center for Interdisciplinary Research (CiiEM), Egas Moniz School of Health & Science, 2829-511 Almada, Portugal; vmachado@egasmoniz.edu.pt (V.M.); iargolinha@egasmoniz.edu.pt (I.A.); jrua@egasmoniz.edu.pt (J.R.); jmendes@egasmoniz.edu.pt (J.J.M.); jbotelho@egasmoniz.edu.pt (J.B.); 2 Department of Biologic and Materials Sciences & Prosthodontics, School of Dentistry, University of Michigan, Ann Arbor, MI 48109-1078, USA; junying@umich.edu

**Keywords:** jaw tracking, mandibular kinematics, prosthetic rehabilitation, digital dentistry, virtual articulator

## Abstract

**Background/Objectives**: To characterize clinical values obtained from a digital jaw tracking system during the functional assessment of mandibular movements in patients undergoing prosthetic rehabilitation. **Methods**: Ten patients undergoing prosthetic rehabilitation were included. Mandibular movements were recorded using the Zebris JMA Jaw Tracking System and measurements including maximum mouth opening, lateral excursions, protrusion, and condylar path length. Descriptive analyses were performed. Quantitative parameters were summarized as mean ± standard deviation and range and compared with normative values reported in the literature. **Results**: Ten participants completed mandibular kinematics registration (8 female, 2 male). Considerable interindividual variability was observed across all parameters. Mean maximum opening was 38.19 ± 10.99 mm (range 22.5–59.6 mm), mean protrusion 5.79 ± 4.47 mm (range 3.0–17.8 mm), mean right lateral excursion 6.56 ± 3.06 mm, and mean left lateral excursion 4.90 ± 2.11 mm. Lateral asymmetry was identified in 5 out of 10 patients. Bilateral condylar path length asymmetry had a mean of 3.59 ± 1.89 mm. **Conclusions**: Values obtained through the jaw tracking system demonstrated considerable interindividual variability in this prosthetic rehabilitation population. These preliminary findings support the need for further research to characterize normative kinematic values in this specific population and to evaluate the clinical impact of integrating jaw tracking data into prosthetic planning workflows.

## 1. Introduction

Contemporary prosthetic rehabilitation requires not only accurate static occlusal relationships but also a comprehensive understanding of dynamic mandibular function. Functional mandibular movements play a critical role in ensuring biomechanical compatibility, occlusal stability, and the long-term success of prosthetic restorations [1].

Recent advances in digital technologies have significantly improved the precision and applicability of virtual articulators, allowing the simulation of occlusal relationships within virtual environments and enhancing treatment planning workflows [2,3,4]. These developments are largely driven by improvements in digital data acquisition, CAD/CAM technologies, and the integration of advanced computational tools, including artificial intelligence, which have contributed to more accurate and efficient clinical procedures [3]. As a consequence, this evolution is paving the way towards a digital dentistry that is more comprehensive and patient-specific-driven [5,6].

Despite these advances, the accurate reproduction of individual mandibular movement remains a major challenge. Conventional and virtual articulators often rely on average anatomical values or simplified motion parameters, which may not reflect patient-specific functional dynamics [7,8]. As a result, important aspects such as movement asymmetries, deviations, and functional variability may not be fully captured. The implementation of fully digital workflows in prosthetic dentistry has further highlighted the importance of integrating both static and dynamic data. These workflows have demonstrated improved precision and reproducibility, particularly in complex rehabilitations [9,10]. Nevertheless, the accurate registration of maxillomandibular relationships remains a critical step.

Digital jaw tracking systems have emerged as a promising solution for recording mandibular kinematics and integrating functional data into digital workflows [11]. These systems employ different technologies—including ultrasonic, optoelectronic, and electromagnetic tracking—to capture three-dimensional mandibular movements with a level of detail not achievable by conventional clinical examination or traditional articulators [11,12].

However, their incorporation into routine prosthetic rehabilitation remains limited and insufficiently explored [12].

It is also important to acknowledge that normative reference values for mandibular movements exhibit substantial variability across populations, depending on factors such as age, sex, malocclusion, dental status, and the presence of temporomandibular disorders [13]. Reported normative ranges for maximum mouth opening typically span 38–50 mm, lateral excursions 8–10 mm, and protrusion 8–12 mm in asymptomatic adults; however, these ranges may differ significantly in patients with partial or full edentulism, altered occlusal vertical dimension, or ongoing prosthetic rehabilitation [13]. This variability underscores the need for patient-specific kinematic assessment rather than reliance on population averages. By recording parameters such as maximum mouth opening, lateral excursions, protrusion, condylar path inclination, and the envelope of function, jaw tracking systems provide individualized kinematic data that may directly influence prosthetic design decisions [13,14,15,16]. Incorporating these parameters into digital workflows may therefore improve the fit, occlusal balance, and longevity of prosthetic restorations, particularly in full-arch rehabilitations or cases with compromised mandibular function [17,18]. For instance, condylar inclination values influence the cusp inclination of posterior restorations, lateral excursion ranges define the required canine and posterior disclusion, and the envelope of function determines the functional boundaries within which restorations must operate [18,19].

Despite increasing availability, evidence on the clinical applicability of jaw tracking in routine prosthetic workflows remains limited. Therefore, this study aimed to characterize mandibular kinematic values recorded by a digital jaw tracking system during the functional assessment of patients undergoing prosthetic rehabilitation, as a preliminary and exploratory step towards understanding the functional variability of this population.

## 2. Materials and Methods

### 2.1. Study Design

A descriptive clinical case series was conducted to evaluate the clinical applicability of a digital jaw tracking system within a prosthetic rehabilitation setting.

No formal sample size calculation was performed, as this study was designed as a preliminary exploratory case series. The sample was determined by convenience, based on patient availability within the clinical setting during the study period. This is consistent with the descriptive nature of the study, which does not aim to establish statistical significance but rather to characterize the feasibility and variability of jaw tracking recordings in a prosthetic rehabilitation.

### 2.2. Participants

A series of participants undergoing prosthetic evaluation and treatment planning at the Dental Clinic at Egas Moniz School of Health and Science were included in this study. All patients were undergoing prosthetic evaluation for a single-unit crown restoration over a natural tooth, representing a dentate population with localized tooth loss. All patients presented with preserved posterior support and were in stable occlusion at the time of recording. None of the included patients had previously received full-arch or removable prosthetic rehabilitation.

Patients were not formally screened using a temporomandibular disorder (TMD) questionnaire; however, those with acute orofacial pain, discomfort interfering with mandibular recording, or a history of neurological or musculoskeletal conditions severely affecting mandibular function were excluded. Overall oral condition was assessed clinically prior to inclusion. Basic demographic characteristics (age and sex) were recorded. All participants provided written informed consent prior to inclusion in the study.

### 2.3. Inclusion Criteria

Patients were eligible if they:

• Were adults aged 18 years or older;
• Were undergoing prosthetic evaluation and/or treatment planning;
• Were able to perform mandibular movements required for functional recording.

### 2.4. Exclusion Criteria

Patients were excluded if they:

• Were unable to perform mandibular movements in a reproducible manner;
• Had acute pain or discomfort that interfered with mandibular movement recording;
• Had a history of neurological or musculoskeletal conditions severely affecting mandibular function.

### 2.5. Jaw Tracking System

Mandibular movements were recorded using the Zebris Jaw Motion Analyzer (Zebris Medical GmbH, Isny im Allgäu, Germany), a digital jaw tracking system based on ultrasonic motion analysis. The system provides individualized functional data, including movement amplitude, trajectory, and symmetry between excursions.

### 2.6. Clinical Procedure

All recordings were performed by a single operator (S.L.), a clinician with specific training in the use of the Zebris JMA system, acquired within the context of a doctoral research programme. Device calibration was performed prior to each recording session following the manufacturer’s standardized protocol, including condyle position definition using the C-Positioner (Zebris Medical GmbH, Isny im Allgäu, Germany) and bite fork setup as specified in the Zebris for Ceramill 4.0.2 software. No formal intra-operator reliability testing was performed, which is acknowledged as a limitation of this preliminary study.

Each participant was instructed to perform the following mandibular movements:

• Maximum mouth opening;
• Right lateral excursion;
• Left lateral excursion;
• Protrusive movement.

The operator visually confirmed proper execution of all movements.

In order to ensure reproducibility and reduce artefacts, each movement was rehearsed three times prior to recording. A single recording was subsequently performed for each movement. The operator monitored the execution of all movements to ensure adequate performance.

### 2.7. Variables Assessed

The jaw tracking recordings were analyzed descriptively with emphasis on the following functional parameters:

Movement amplitude;

Movement trajectory;

Symmetry between right and left excursions;

Presence of deviations during opening or closing;

General clinical interpretability of the recorded movement pattern.

The focus of the study was not on therapeutic outcomes, but rather on the feasibility and usefulness of the system in the functional evaluation of patients undergoing prosthetic rehabilitation.

### 2.8. Data Analysis

Data were analyzed descriptively. Quantitative values were summarized using mean, standard deviation, minimum, and maximum. Qualitative assessment focused on the visualization and interpretation of mandibular movement patterns and on the integration of jaw tracking data into the prosthetic planning workflow.

### 2.9. Ethical Considerations

All procedures were performed in accordance with the ethical standards of the institution and with the principles of the Declaration of Helsinki. Written informed consent was obtained from all participants before recording. The study protocol was approved by the local ethics committee (Egas Moniz Ethics Committee under reference number 1409, approved on 26 June 2024).

## 3. Results

### 3.1. Characteristics of Participants

Ten patients undergoing prosthetic rehabilitation were included in this case series (8 female, 2 male). The average age was 48.8 ± 16.7 years old). The Zebris JMA system successfully recorded mandibular kinematics in all cases, demonstrating consistent clinical applicability across the sample. All recordings were performed using the same configuration: C-Positioner for condyle definition, REF1960430 Alignment Fork, and Camper Plane (CE) as the reference plane, with software version Zebris for Ceramill 4.0.2. Individual patient data are presented in Table 1.

As shown in Table 2, mean maximum mouth opening (38.19 ± 10.99 mm) approximated the lower limit of the normative range (38–50 mm). Mean protrusion (5.79 ± 4.47 mm) and mean lateral excursions (right: 6.56 ± 3.06 mm; left: 4.90 ± 2.11 mm) were below the corresponding normative reference ranges. No established normative reference values exist for condylar path length parameters.

### 3.2. Protrusion and Lateral Excursion Movements

Descriptive statistics for all protrusion and lateral excursion movement parameters are presented in Table 2. Considerable interindividual variability was observed across all parameters.

Recorded values were compared with widely reported normative reference ranges for mandibular movements in healthy adults (mouth opening: 38–50 mm; lateral excursions: 8–10 mm; protrusion: 8–12 mm), as cited in Rahman et al. (2022) [13], which in turn reflected values consistently reported in the literature for asymptomatic populations.

Maximum mouth opening ranged from 22.5 mm to 59.6 mm, with a mean of 38.19 ± 10.99 mm. Four patients (A, E, G, J) presented values below the normative threshold of 38 mm as defined by Rahman et al. (2022) [13], with the lowest value recorded in patient G (22.5 mm).

Protrusion range was from 3.0 mm to 17.8 mm, with a mean of 5.79 ± 4.47 mm. Most patients (8/10) presented values below the normative range of 8–12 mm.

Right lateral excursion ranged from 0.3 mm to 10.1 mm (mean 6.56 ± 3.06 mm), and left lateral excursion ranged from 0.7 mm to 7.1 mm (mean 4.90 ± 2.11 mm), both below the normative range of 8–10 mm. The most markedly reduced values were observed in patient J (0.3 mm right; 0.7 mm left), which may reflect a functional limitation in the context of prosthetic rehabilitation.

Lateral symmetry, defined as a difference of ≤2 mm between right and left excursions, was observed in 7 out of 10 patients. The remaining three patients showed asymmetric lateral excursions, with differences ranging from 2.6 mm to 6.2 mm.

**Figure 1:**
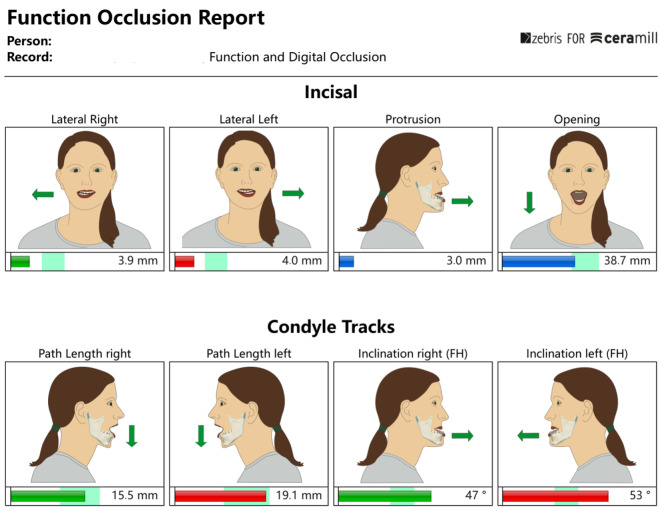
Representative output of the Zebris JMA system (Function Occlusion Report, illustrative case). The upper panel displays the incisal movement recordings: lateral right (3.9 mm), lateral left (4.0 mm), protrusion (3.0 mm), and maximum mouth opening (38.7 mm), each represented with a directional indicator and a colour-coded bar relative to the reference range. The lower panel shows the condyle track parameters: right path length (15.5 mm), left path length (19.1 mm), right inclination (47°), and left inclination (53°). Colour coding indicates proximity to reference ranges (green: within range; red: outside range).

**Figure 2:**
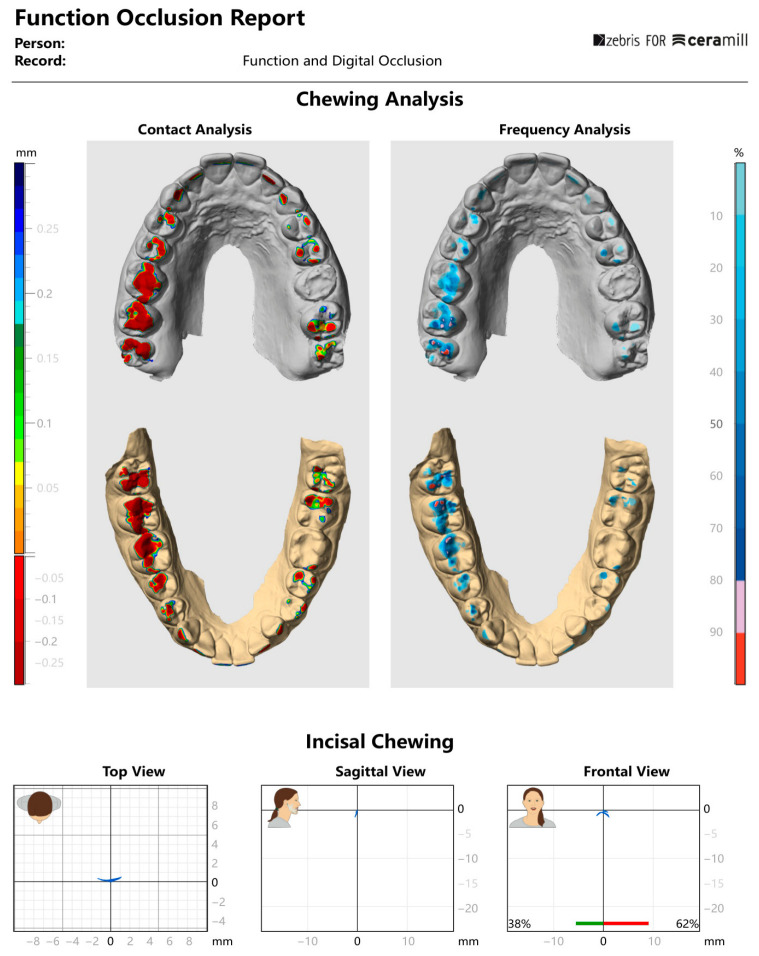
Chewing analysis output from the Zebris JMA system (illustrative case). The Contact Analysis (**left**) displays the spatial distribution and magnitude of occlusal contacts during chewing cycles, colour-coded by contact depth (mm). The Frequency Analysis (**right**) represents the percentage of chewing cycles during which each tooth area was contacted. The lower panel shows the incisal chewing path in three planes (top, sagittal, and frontal views), illustrating the envelope of the chewing cycle and the left–right distribution of chewing activity. The colour scale on the left (mm) represents contact depth in the Contact Analysis, ranging from red (deepest contact, −0.25 mm) to blue (shallowest contact, +0.25 mm). The colour scale on the right (%) represents contact frequency in the Frequency Analysis, ranging from red (highest frequency, 90%) to light blue (lowest frequency, 10%).

**Figure 3:**
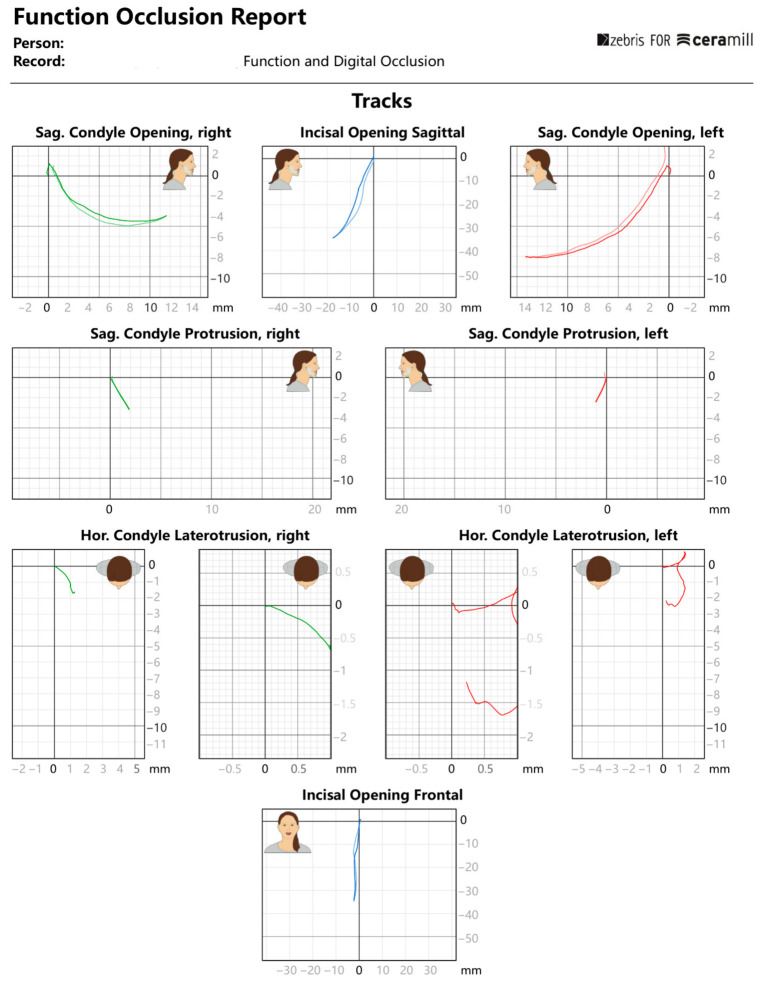
Detailed condyle track recordings from the Zebris JMA system (illustrative case). Sagittal condyle opening tracks (right and left), incisal opening paths (sagittal and frontal), sagittal condyle protrusion tracks (right and left), horizontal condyle laterotrusion tracks (right and left), and the incisal opening frontal path are displayed. These recordings allow qualitative assessment of movement trajectories, symmetry between sides, and detection of deviations or irregularities during mandibular movements.

The qualitative output provided by the Zebris JMA system, including movement trajectories, condyle tracks, chewing analysis, and incisal opening paths, is illustrated for a representative case in Figure 1, Figure 2 and Figure 3. These recordings demonstrate the type of functional information available through the system and support its feasibility for qualitative assessment in prosthetic rehabilitation patients.

### 3.3. Condyle Tracks

Condylar path length was recorded bilaterally in all patients. Right path length ranged from 9.3 mm to 22.2 mm (mean 15.44 ± 4.27 mm), and left path length ranged from 10.3 mm to 25.5 mm (mean 15.33 ± 3.97 mm). Bilateral condylar path length asymmetry, expressed as the absolute difference between right and left values, ranged from 0.7 mm to 6.1 mm (mean 3.59 ± 1.89 mm), indicating that most patients presented some degree of bilateral condylar asymmetry in path length.

No established normative reference values exist for condylar path length, as this parameter is inherently dependent on the magnitude of mouth opening and therefore not directly comparable across individuals. Accordingly, path length data were analyzed descriptively and in terms of bilateral asymmetry only.

Clinically, reduced protrusion may have implications for anterior restoration design decisions, as jaw tracking data has been shown to directly inform restoration morphology and occlusal parameters [18,19].

From a clinical perspective, reduced lateral excursions may also have implications for prosthetic planning decisions, as jaw tracking data has been shown to directly inform dynamic occlusal surface morphology and restoration design [18,19].

## 4. Discussion

The present exploratory case series aimed to characterize mandibular kinematic values recorded with a digital jaw tracking system in patients undergoing prosthetic rehabilitation. The main finding was considerable interindividual variability across all recorded parameters, including movement amplitude, lateral symmetry, and condylar path length. While these findings support the feasibility of integrating jaw tracking into prosthetic evaluation workflows, they should be interpreted with caution given the significant methodological limitations of this study, including the small and convenience-based sample, the absence of intra-operator reliability testing, the lack of clinical outcome data, and the descriptive nature of the analysis.

These results highlight the potential value of digital jaw tracking systems for providing objective and individualized functional data that may complement conventional prosthetic assessment methods. The integration of digital technologies into prosthodontics has led to more precise and reproducible treatment planning approaches [9,10]. However, the incorporation of dynamic mandibular data into these workflows remains a key challenge. Jaw motion tracking systems provide a potential solution by allowing the real-time recording of mandibular kinematics. These systems are based on different technologies, including ultrasonic and optoelectronic tracking, enabling three-dimensional analysis of mandibular movements. Previous studies have demonstrated that jaw tracking systems present acceptable levels of accuracy and reproducibility, supporting their use in functional analysis [14,15,16].

Previous studies using jaw tracking systems in clinical populations have similarly reported interindividual variability in kinematic values and, in populations with functional limitations, values below normative references [13,15,16]. These findings suggest that reduced kinematic values may be a characteristic feature of patients undergoing dental rehabilitation, rather than an artifact of the recording system.

In addition, the integration of jaw tracking into digital workflows has been shown to improve the registration of maxillomandibular relationships, allowing a more patient-specific approach for prosthetic planning [17]. This contributes to the concept of a “virtual patient,” in which both static and dynamic parameters are considered [5]. The ability to record the envelope of function has also been highlighted as a relevant factor in prosthetic design, as it defines the functional boundaries within which restorations must operate [18]. Furthermore, recent evidence indicates that digital jaw motion tracking systems can substantially enhance the accuracy of dynamic occlusal surface morphology and condylar inclination measurements, providing functional details that are not captured by conventional or virtual articulators [19].

In the present study, jaw tracking allowed individualized visualization of mandibular dynamics in all ten patients. Considerable interindividual variability was observed, with maximum mouth opening ranging from 22.5 to 59.6 mm and lateral excursions ranging from 0.3 to 10.1 mm, highlighting the limitations of relying on average normative values in prosthetic planning. Although several patients presented protrusion and lateral excursion values below commonly reported normative references, all individual measurements remained within the observed sample ranges. These findings should be interpreted in the context of the exploratory nature of this study and the functional variability expected in a prosthetic rehabilitation population. Lateral asymmetry was identified in 3 out of 10 patients, a finding that would not have been detectable through conventional clinical assessment alone.

Despite these advantages, the use of jaw tracking systems in clinical practice remains limited. A recent systematic review highlighted the growing interest in these technologies while emphasizing the need for further clinical validation [12]. Systems such as the Zebris JMA Jaw Tracking System allow the acquisition of patient-specific functional data, which may help overcome the limitations of conventional and virtual articulators. These articulators, although increasingly sophisticated, still rely on simplified movement simulations and average values [7,8]. Mandibular movement asymmetries, deviations and functional variability are clinically relevant factors that limit the accurate reproduction of individual mandibular function and may impact individualized prosthetic planning. These functional features, such as the envelope of function, cannot be captured using conventional and virtual articulators, which depend on average anatomical parameters or simplified motion pathways [7,8].

Bilateral condylar path length asymmetry was identified in most patients (mean difference 3.59 ± 1.89 mm). As no established normative values exist for this parameter, these findings are reported descriptively and should not be interpreted as indicative of pathology or clinical dysfunction.

Although the small sample size limits the generalizability of the findings, this case series was not designed to establish normative data but rather to explore the feasibility of recording and characterizing mandibular kinematic values in a real prosthetic rehabilitation setting using a digital jaw tracking system. The preliminary and exploratory nature of this study must be explicitly acknowledged when interpreting these results. The convenience-based sampling method, the absence of formal intra-operator reliability testing, the lack of a control group, the absence of follow-up data, and the absence of clinical outcome data and of a control group of asymptomatic individuals with ideal occlusion all represent significant limitations that must be explicitly acknowledged when interpreting these results. The non-equal distribution of patients by sex (8 female, 2 male) may also introduce bias, as mandibular kinematic parameters are known to differ between males and females [13]. Furthermore, the comparison with normative reference ranges should be regarded as contextual rather than conclusive, given that the cited values represent general population averages and may not be directly applicable to a prosthetic rehabilitation population. No direct correlation was established between the recorded kinematic values and prosthetic treatment decisions or outcomes.

The absence of prosthodontic diagnosis-specific normative kinematic values represents a critical gap in the literature, as the reference ranges currently used were established in asymptomatic dentate adults and may not be directly applicable to patients undergoing prosthetic rehabilitation [13,16]. Future studies should aim to stratify normative kinematic data by prosthetic diagnosis, occlusal status, and treatment stage.

From a clinical relevance perspective, the findings of this study suggest that digital jaw tracking can provide functional information that goes beyond what is routinely captured in prosthetic examination [11,12]. Reduced lateral excursions and protrusion, as observed in the majority of patients in this series, may have implications for prosthetic planning decisions, as jaw tracking data has been shown to directly inform dynamic occlusal surface morphology and restoration design [18,19]. While this study does not establish a causal relationship between kinematic findings and prosthetic outcomes, it demonstrates that jaw tracking data can reveal clinically relevant functional features that are not detectable by conventional assessment alone.

These findings nonetheless provide a preliminary descriptive foundation for future controlled studies with larger samples, repeated measurements, and clinical outcome measures. Future research should focus on evaluating the impact of incorporating mandibular motion data into prosthetic design, particularly in relation to occlusal adjustment, restoration longevity, and patient comfort.

## 5. Conclusions

This exploratory case series suggests that digital jaw tracking systems can identify substantial interindividual variability in mandibular kinematics among patients undergoing prosthetic rehabilitation. These findings highlight the potential value of objective functional assessment in supporting individualized prosthetic treatment planning. Nevertheless, larger controlled studies incorporating clinical outcome measures are required to establish the clinical relevance of these observations and to determine the role of digital jaw tracking in evidence-based prosthetic workflows.

## Figures and Tables

**Table 1:** Clinical data collection (Case Series, *n* = 10).

Identification		Protrusion and Lateral Movement (mm)					Condyle Tracks		
Patient	Sex	Max. Opening (mm)	Protrusion (mm)	Lat. R (mm)	Lat. L (mm)	Symmetric? (R vs. L)	Path Length R (mm)	Path Length L (mm)	Path Length Diff. (mm)
A	F	34.1	3.8	3.2	3.6	Yes	22.2	17.2	5.0
B	F	37.3	3.7	8.1	7.1	Yes	12.9	18.0	5.1
C	F	59.6	4.0	5.1	6.4	Yes	13.5	17.9	4.4
D	M	38.3	6.6	4.5	5.8	Yes	13.2	11.1	2.1
E	M	28.5	5.0	9.7	7.1	No	16.3	18.6	2.3
F	F	38.7	3.0	3.9	4.0	Yes	15.5	19.1	3.6
G	F	22.5	5.0	8.5	6.0	No	9.3	11.2	1.9
H	F	46.0	8.0	6.1	6.5	Yes	22.0	25.5	3.5
I	F	39.5	17.8	10.1	3.9	No	10.5	14.5	4.0
J	F	28.9	3.0	0.3	0.7	Yes	11.0	10.3	0.7
Reference values	—	38–50 mm *	8–12 mm *	8–10 mm *	8–10 mm *	N/A †	N/A †	N/A †	N/A †
Descriptive Statistics									
Mean	—	37.34	5.99	5.95	5.11	7/10	14.64	16.34	—
SD	—	10.32	4.44	3.14	2.03	n/a	4.47	4.67	—
Minimum	—	22.50	3.00	0.30	0.70	n/a	9.30	10.30	—
Maximum	—	59.60	17.80	10.10	7.10	n/a	22.20	25.50	—
Valid n	—	10	10	10	10	10	10	10	—

Lat. R: right lateral excursion; Lat. L: left lateral excursion; Diff.: absolute difference between right and left path length; SD: standard deviation; n/a: not applicable; —: not available. Symmetric: defined as a difference ≤ 2 mm between right and left lateral excursions. Reference values (Rahman et al., 2022 [13]): opening 38–50 mm; protrusion 8–12 mm; lateral excursions 8–10 mm.

**Table 2:** Descriptive statistics of mandibular kinematic parameters recorded with the Zebris JMA system (*n* = 10).

Parameter	Mean ± SD	Range	Reference Values
Max. Opening (mm)	38.19 ± 10.99	22.5–59.6	38–50 mm *
Protrusion (mm)	5.79 ± 4.47	3.0–17.8	8–12 mm *
Lateral Excursion R (mm)	6.56 ± 3.06	0.3–10.1	8–10 mm *
Lateral Excursion L (mm)	4.90 ± 2.11	0.7–7.1	8–10 mm *
Path Length R (mm)	15.44 ± 4.27	9.3–22.2	N/A †
Path Length L (mm)	15.33 ± 3.97	10.3–25.5	N/A †
Path Length Diff. (mm)	3.59 ± 1.89	0.7–6.1	N/A †

* Reference values: Rahman F, Femiano F, Louis PJ, Kau CH. Medicina 2022;58(6):738 [13]. SD: standard deviation. † No established normative reference values exist for condylar path length, as this parameter is inherently dependent on mouth opening magnitude and is therefore not directly comparable across individuals.

## Data Availability

The data are not publicly available due to privacy and ethical restrictions, as they contain sensitive patient information collected under informed consent for research purposes only.

## References

[1] Abduo J., Lyons K. (2012). Clinical considerations for increasing occlusal vertical dimension: A review. Aust. Dent. J..

[2] Lobo S., Argolinha I., Machado V., Botelho J., Rua J., Li J., Mendes J.J. (2025). Advances in digital technologies in dental medicine: Enhancing precision in virtual articulators. J. Clin. Med..

[3] Alghauli M.A., Aljohani W., Almutairi S., Aljohani R., Alqutaibi A.Y. (2025). Advancements in digital data acquisition and CAD technology in dentistry: Innovation, clinical impact, and integration of AI. Clin. eHealth.

[4] Mangano F., Gandolfi A., Luongo G., Logozzo S. (2017). Intraoral scanners in dentistry: A review of the current literature. BMC Oral Health.

[5] Özdemir G., Albayrak B., Yuzbasioglu E., Olcer Us Y. (2021). Virtual articulators, virtual occlusal records and virtual patients in dentistry. J. Exp. Clin. Med..

[6] Güth J.F., Keul C., Stimmelmayr M., Beuer F., Edelhoff D. (2013). Accuracy of digital models obtained by direct and indirect data capturing. Clin. Oral Investig..

[7] Solaberrieta E., Minguez R., Barrenetxea L., Etxaniz O., Otegi J.R. (2015). Comparison between a conventional and a virtual articulator for the analysis of dental occlusion: A review. J. Prosthet. Dent..

[8] Abduo J., Rasaie V. (2025). Digital workflows in prosthodontics. Aust. Dent. J..

[9] Joda T., Zarone F., Ferrari M. (2017). The complete digital workflow in fixed prosthodontics: A systematic review. BMC Oral Health.

[10] Mahato M., Hota S., Jain A., Dutta D., Bhushan P., Raut A. (2024). Comparison of conventional and digital workflows in the fabrication of fixed prostheses: A systematic review. Cureus.

[11] Jakubowska S., Szerszen M., Kostrzewa-Janicka J. (2023). Jaw motion tracking systems: Literature review. Protet. Stomatol..

[12] Tafuri G., Santilli M., D’Addazio G., Murmura G., Traini T., Femminella B., Caputi S., Sinjari B. (2026). Jaw tracking system in digital dentistry: A systematic review. Int. J. Prosthodont..

[13] Rahman F., Femiano F., Louis P.J., Kau C.H. (2022). An evaluation of jaw tracking movements in patients with total joint replacements versus a control group. Medicina.

[14] Nagy Z., Mikolicz A., Vag J. (2023). In vitro accuracy of a novel jaw-tracking technology. J. Dent..

[15] Grande F., Lepidi L., Tesini F., Acquadro A., Valenti C., Pagano S., Catapano S. (2024). Investigation of the precision of a novel jaw tracking system in recording mandibular movements: A preliminary clinical study. J. Dent..

[16] Revilla-León M., Zeitler J.M., Fry E., Kois J.C. (2025). Digital workflow to measure the mandibular range of motion using different jaw tracking technologies. J. Prosthet. Dent..

[17] Feng Y., Zhan L., Sun X., Li J., Liu W. (2023). A fully digital workflow to register maxillomandibular relation using a jaw motion tracer for fixed prosthetic rehabilitation: A technical report. J. Esthet. Restor. Dent..

[18] Kois J.C., Zeitler J.M., Revilla-León M. (2025). Use of an optical jaw tracking system to capture the envelope of function when designing interim and definitive prostheses: A dental technique. J. Prosthet. Dent..

[19] Saygılı S., Özcan-Sezgin A., Aktosun A., Bilgen B., Sülün T. (2025). Impact of digital jaw tracking systems on dynamic occlusal surface morphology and condylar inclination measurements. J. Adv. Prosthodont..

